# Introducing Care 4.0: An Integrated Care Paradigm Built on Industry 4.0 Capabilities

**DOI:** 10.3390/ijerph16122247

**Published:** 2019-06-25

**Authors:** Chaloner Chute, Tara French

**Affiliations:** 1 Digital Health and Care Institute, University of Strathclyde, Glasgow G1 1RD, UK; 2 The Glasgow School of Art, Forres IV36 2SH, UK

**Keywords:** care 4.0, industry 4.0, health 4.0, integration, trust, co-design, person-centered, distributed, community, care

## Abstract

Western developed health and care policy is shifting from a patriarchal medical model to a co-managed and integrated approach. Meanwhile, the fourth industrial revolution (Industry 4.0) is transforming manufacturing in line with the digital consumer revolution. Digital health and care initiatives are beginning to use some of the same capabilities to optimize healthcare provision. However, this is usually limited to self-management as part of an organization-centric delivery model. True co-management and integration with other organizations and people is difficult because it requires formal care organizations to share control and extend trust. Through a co-design lens, this paper discusses a more person-centered application of Industry 4.0 capabilities for care. It introduces ‘Care 4.0’, a new paradigm that could change the way people develop digital health and care services, focusing on trusted, integrated networks of organizations, people and technologies. These networks and tools would help people co-manage and use their own assets, in the context of their own care circle and community. It would enable personalized services that are more responsive to care needs and aspirations, offering preventative approaches that ultimately create a more flexible and sustainable set of integrated health and social care services that support meaningful engagement and interactions.

## 1. Introduction

There is a well understood sustainability crisis affecting Western European health and care economies [1]. A range of issues contribute to this (e.g., the ever-increasing costs of treatment and medicine), however one of the critical trends is around the ageing population and an increasingly fragmented informal care dynamic caused in part by societal changes and the forces of globalization [2]. A significant part of the response to these challenges is the strengthening of community care which is common across Europe [3]. In Scotland, policy and strategy are driving towards more preventative, co-managed, integrated and community-based care, with digital technology seen as a key asset to deliver change at scale [4]. The Scottish mode of transformation reflected in the emerging digital health and care market is accelerating, valued at $86.4 billion in 2018 and projected to be valued at $504.4 billion by 2025 [5]. 

The Digital Health and Care Institute (DHI) is one of many innovation centers, incubators, think tanks, and accelerators which have been setup to support both health and care transformation and economic development. DHI is distinct in its whole system approach, which is underpinned by co-design with those providing and using health and social care services [6]. The market has notably matured in the five years of DHI’s existence and is now on the cusp of a significant shift as service design and technology strategies look to develop whole system models based on existing large-scale deployment of digital capabilities in other sectors [4,7,8]. In parallel, consumerism is now escalating in a broader digital market economy. By 2020, 90% of UK adults will use a smart phone and 50% will have an average of four online media subscription services [9]. People are becoming increasingly accustomed to choice and use of flexible, dynamic services that stay relevant and useful within a rapidly changing world. 

These two forces—(i) strategic, and systemic service redesign enabled by technology and (ii) the emerging digital consumer, have intersected during the early stages of the fourth industrial revolution (Industry 4.0). Industry 4.0 provides the capability to connect everything to the internet, generating data across the whole process and using advanced analytics to support completely new products and service models. This capability allows for decentralization and/or distribution of power and capability throughout the value chain.

It is likely that Industry 4.0 capabilities (4.0 toolkit) will be initially applied to health and social care systems following the dominant organization-centric discourse. This risks isolating the user as an independent consumer being managed by, and passively consuming from, a formal health or social care service. Within this approach there may be the possibility of self-management by that person, but as a fixed part of the organization’s top-down delivery model. 

The market must be stimulated instead, to work towards emergent empowerment and co-management models targeted by government policy. If people can be involved in co-producing their own, asset-based service models, this would allow the 4.0 toolkit to re-integrate communities and rebuild trust and interdependency among people. This is what Nesta, a UK innovation foundation, calls for in its plea to ‘make the fourth industrial revolution good’, i.e., for civil society to shape the fourth industrial revolution to be more productive for society than previous revolutions—which displaced and disenfranchised millions and focused energy and resources on environmental consumption, profit, and war [10].

This paper provides an overview of trends in Scottish health and care policy alongside the key integrated and person-centered care themes emerging from the co-design and design research within DHI to date. It goes on to summarize an emerging toolset from the Fourth Industrial Revolution (Industry 4.0) that is transforming other sectors and then demonstrates the use of this ‘4.0’ toolset for optimization of traditional healthcare provision (Health 4.0). It then argues that Health 4.0 alone will not deliver the services targeted in policy and proposes a new paradigm, ‘Care 4.0’, to enable integrated, person-centered care services. 

## 2. Health and Care Policy

**Figure 1:**
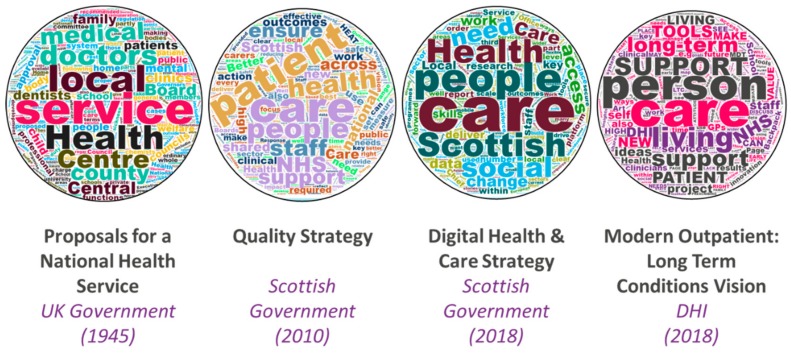
Visualizing the shifts in language in Scottish health and social care policy [4,11,12,13].

Across the UK, the practices and premise of health and social care are shifting to enable a system that supports integrated and person-centered care. In recent years, the Scottish health and social care policy context has been moving from a patriarchal medical model, with resources concentrated in hospitals, to an integrated, co-managed, and person-centered model based in the community. This shift aligns with policies across Europe to strengthen the role of primary and community care in support of person-centered, sustainable services [3]. The British language of policy and practice is changing in tandem (see Figure 1), shifting from terms like patient, health, medical, doctor; to person, living, care, and support. 

Health and care research, innovation, and investment expenditure are not yet shifting with policy, remaining primarily focused on optimizing medicine, treatment of disease, and modernizing hospital and tele-medicine models. Interventions by the medical community, such as Realistic Medicine [14], have provided vision, but as yet there are few examples of putting this into practice at scale. 

Digital health and care is heralded as a way of supporting this shift whilst also creating a more sustainable system. This expectation is predicated on improved data sharing, advanced analytics, and automation. Scotland is investing in this through the Digital Health and Care Strategy to support the development of more personalized and predictive services [4]. 

However, these benefits will not manifest if resources continue to focus on transactional relationships between citizens and health and social care systems. This is because in order to personalize and predict, systems must be informed by the person’s own context. Therefore, it is proposed that the digital health and social care toolset must help systems by understanding people’s lived experience. This includes their current health and wellbeing activity, health, and social care interventions in a broader life and environmental context and any resulting holistic outcomes. The toolset must be able to balance the system’s need for information with a personal need for trust and the ability to connect to informal care circles and communities. It must be able to use any formal or informal assets to help sustain the engagement, care interactions, and experiences on a co-managed basis.

## 3. Consolidating Learning from Co-Design in Scotland

Over the past five years, the academic design research team who are part of DHI have undertaken over 20 design-led projects involving people with lived experience, practitioners across a range of health, social care and third sector organizations, academic subject experts, and industry partners. Core to these projects has been the practice of co-design which supports the involvement of end users in the design process by valuing people as ‘experts of their experience’ [15]. Co-design empowers people with diverse experiences to come together and conceptually explore, develop, and create their own ideas to respond to a situation or design task [16]. Within DHI, co-design practice is employed to support participants to explore and prototype person-centered digital solutions to health and social care challenges. Projects have explored a range of challenges including data sharing across health and social care systems, reimagining outpatient services to support people with long term conditions, and public health topics such as promoting breastfeeding [17].

The outputs of co-design have resulted in a body of knowledge of the role of digital technology in health and social care in Scotland evident through the findings, insight, and concepts generated from these projects. For the purposes of this paper, the following learnings are considered key to informing future developments in this area.

### 3.1. The Role of Digital Technology in Health and Care is Enabling

One of the key learnings across all projects is the way in which technology should enable person-centered care, whether this is from the perspective of those providing or those receiving care and services. Technology needs to enable the right care at the right time through providing access and ease of use for citizens to have control of interactions with systems and services, helping them to activate services on their own terms. Supporting an asset-based [18,19] rather than deficit model of care allows the wider system to be responsive and proactive, rather than reactive, by adapting to the changing needs of the person, providing flexible access and engagement with services. Developing technology that ‘enables’ the provision and receipt of care also alleviates fears that technology will replace human interaction where it is most valued and appropriate. This has previously been described as creating a ‘community of care’ [20] where technology is not a replacement of services but provides a way to facilitate connections, redressing the balance between this and the burden of time consuming, organization-centric, risk management activities. 

### 3.2. The Person as the Point of Integration

A prominent recurring challenge across all areas of co-design has been the difficulty of sharing information across systems, services, and access to information for citizens [21,22]. In Scotland, there are twenty-one health boards and thirty-one health and social care partnerships within and across the different health boards. Within each of these organizations are then further silos, for example the largely separate hospital and primary care systems, or the specialisms within the outpatients system. This is further compounded by an additional layer of services such as community pharmacies and other high street National Health Service (NHS) contractors, as well as thousands of third sector organizations providing diverse, localized services. All of these care providers have different systems for collecting data which leads to silos in terms of who has access to information. Further, these systems do not communicate with each other which can lead to people interacting with services having to ‘tell their story’ repeatedly to the many different care providers. In this regard, Scotland mirrors similar degrees of organizational complexity found in all health and care systems globally. To resolve this challenge, it is proposed that the person themselves should be the point of integration, allowing people to activate services across health, social care, third sector and communities by sharing their information to enable seamless care interactions. Enabling people to hold their own health and social care information would overcome the challenges of systems data sharing as well as reduce the amount of times people need to repeat information about their own ‘health story’ [23]. In addition, this would enable person-centered care by building services that respond to personal care needs rather than react to the results of unmet need. This would allow people to have more meaningful dialogue with health and social care professionals supporting shared decision making, allow people to better navigate and activate health, social care, third sector and community services that are most appropriate, and allow people to self-manage by understanding patterns in combinations of clinical and personal data [13]. 

### 3.3. Building Trust in Systems and Across People

A key implication from the co-design learning is the need to further explore and understand how to build trust in the context of digital health and social care. This is not only in relation to trust in systems and information (human–system) but also in relation to people having control of sharing information (human–human through system). The co-design research in DHI has not explicitly explored trust in the context of digital health and social care, however, indications from one project exploring the concept of a person-owned data store suggested that participants were in favour of pragmatic sharing of information where benefits outweighed risks in terms of gaining better outcomes through sharing information [23]. Placing control of health and social care information with the person (point of integration) supports agency and enables shared decision making. However, given the wide-ranging interest and differing levels of health literacy, and given the scope for exploitation or error, there is a need to explore the way in which trust is built between people, data and systems from a range of perspectives. 

### 3.4. Enabling a Culture of Innovation

Introducing and deploying technology in the health and social care context involves several considerations for the way in which technology is implemented, adopted, and leads to change and impact. The co-design learnings identified have implications for the wider system and workforce culture which are critical to ensuring technology is truly enabling. Supporting a culture of innovation across the system and workforce is key to ensuring that the system and workforce are ‘ready’ for change. This emphasizes the need to ensure that technology is designed ‘with’ not ‘for’ in order to understand the impacts on existing processes, services, and systems, as well as attitudes, behaviours, and ways of working. Involving people who are likely to be the ‘end-users’ of technology in the design process helps to foster a culture of innovation by giving people permission and a safe space to generate ideas and critically reflect and evaluate potential solutions [24]. This aligns to the Scottish Digital Health and Social Care strategy which seeks to create a permissive culture in order to rebalance approaches to risk [4].

Increasing service pressures are now creating the demand for digital health and care capabilities to be deployed at a far larger scale. This prompts analysis of global trends in the way technology supports new business and service model change at scale.

## 4. Industry 4.0: The Fourth Industrial Revolution

**Figure 2:**
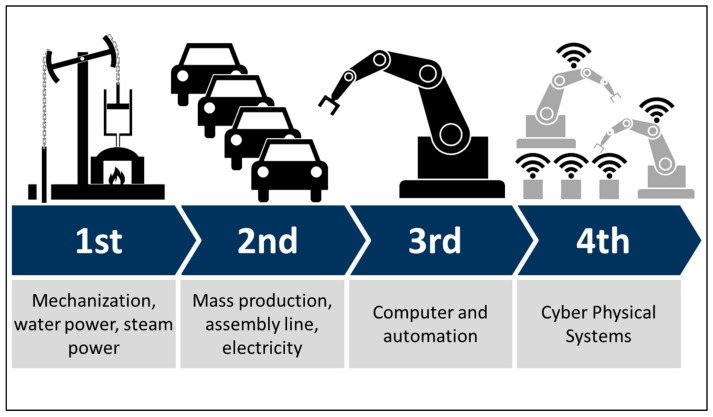
Summary of characteristics of the industrial revolutions [25].

The Fourth Industrial revolution, like the others before, was born in manufacturing connecting everything to the internet, generating data across the whole process, and using advanced analytics to support completely new products and service models (Figure 2). 

Focusing first on a Smart Factory, the initial premise was to create a better connected production line and generate decision support to workers to keep the line running at maximum efficiency, removing waste, and minimizing costs. Over time the quality and configuration of products could be changed more flexibly. Connectivity then expanded outside of the factory to other factories to optimize stock levels and ordering. Industry 4.0 now includes full value chain monitoring, including how the product is used, delivered, and maintained, creating a feedback loop to design and build and target products better in the future, keeping businesses competitive in a fast-evolving marketplace [26]. This revolution must meet needs at a massive industrial scale and is projected to be worth $214 billion by 2023 [27].

The Industry 4.0 toolset includes [28]:

- Cyber Physical System (CPS)—a connected, automated device, capable of learning from and effecting the physical environment, that is intelligent and responsive and can act independently (e.g., self-diagnosing problems) or interdependently with humans or other CPSs to achieve efficiencies or resolve issues.
- Internet of Things (IOT)—the network over which CPSs can connect to the internet and to each other in a secure, auditable manner.
- Internet of Services (IOS)—when devices are networked over the IOT, new services focusing on logistics, intelligence, automation and prediction are possible.
- Smart Factory—the combination of cyber-physical systems and humans, connected through the internet of things with support from the internet of services, monitor production processes, and make de-centralized decisions as part of an interdependent network. The factory management is orchestrated via smart enterprise resource planning (SERP) systems and supported by human and virtual agents to develop product that is responsive in real time to demand, market conditions, and value chain (e.g., logistics) feedback.

Table 1 depicts an example of the toolset applied within the context of manufacturing and provides a baseline capability map for an overarching ‘4.0’ toolset which can be applied to different sectors and delivery models. Subsequent sections will consider other applications of this toolset in emerging next generation models of health and social care. For these discussions the ‘smart factory’ has been jointly labelled a ‘virtual agent’ to allow the concept to better cross sectors. The largely automated intelligent oversight of an interconnected system is a ‘smart factory’ in a manufacturing context, but in a care setting it is more likely to be a virtual assistant that translates between the system and the people within it, wherever they are. 

Since the World Economic Forum’s action on Industry 4.0 [29], many people have attempted to respond to the early challenge and opportunity [30,31]. The concept is still maturing, with discussion around the difficulty of defining this complex web of technologies and principles [32]. Contributors instead characterize Industry 4.0 through several different lenses. For example, arguing that it can only be fully characterized by the changing the way organizations, business models and markets work to optimize the benefits from the technology [32]. A second characterization looks at how the relationships between actors differs from previous revolutions—with the first and second revolutions operating as a centralized network, the third revolution as a decentralized network with multiple, powerful hubs, while the fourth revolution will constitute a distributed network made up of interconnected nodes with equal power [32]. The implications are that given the way the connected value chain reaches well beyond the physical confines of a factory, these technologies will change how society organizes itself, with power and assets redistributed (though not necessarily for societal good).

## 5. Health 4.0: Industry 4.0 Applied to Healthcare

Application of the 4.0 toolset in medicine has been characterized as ‘Health 4.0’ by some [33] but encompasses many related narratives around the use of the IOT in medical care. These approaches tend to focus on the patient in a hospital or other clinical setting, optimizing and tailoring treatment for better clinical outcomes, and to stabilize a stretched healthcare system. As an extension of traditional telehealth initiatives, a new generation of 4.0 based technologies and services continue to grow, attracting large amounts of investment, research, and innovation funds globally [34,35].

The literature around 4.0 capabilities in healthcare is limited, with most of the contributions provided through grey literature market analysis [36,37,38]. Within this, use-cases tend to focus on the Industry 4.0 toolkit applied to optimize existing medical approaches. For example, a medical device can now be manufactured better and cheaper, tracked through supply chain and transport systems, calibrated and supported remotely, connected to triage / diagnostic systems, and the whole process can produce data to optimize how the device is created, used, and maintained, and the existing service improved [39,40]. Note however, that this does not necessarily equate to improved user experience [41].

Thuemmler and Bai [33] state that “The aim of Health 4.0 is to allow for progressive virtualization in order to enable the personalization of health and care next to real time for patients, professionals and formal and informal carers. The personalization of healthcare will be achieved through the massive use of cyber physical systems (CPS), (edge) cloud computing, the internet of everything including things, services and people and evolving mobile communication networks (5G)”. They go on to propose the transfer into a healthcare domain of six Industry 4.0 design principles first set out by [42]. These are:

- Interoperability—networks of people and machines able to communicate via common standards-based data exchange infrastructure.
- Virtualization—thanks to the interoperability of many context aware devices it would then be possible to create a virtual copy of the world to assist the orchestration of the production process.
- Decentralization—moving from the production line mass replication model to one in which autonomous decisions can be made, linking customers and market data into the production process, to allow for mass customization.
- Real-Time Capability—if all parts of the value chain are connected, then the overall model will be able to change in real time.
- Service Orientation—a shift away from products to the use of data to create new services that are more responsive to changing market needs.
- Modularity—moving to a more flexible and agile way of configuring production capabilities, being able to rearrange loosely coupled modules to respond to changing needs.

Addressing the life critical and risk averse nature of healthcare through focusing on smart inhalers and pharmaceuticals in a 4.0 context to enhance the feedback loop between the system, professional, and patient for improved medicine adherence and outcomes, Thuemmler and Bai offer a seventh design principle specifically for Health 4.0:

- Safety, security, resilience—these concepts are “anchored within the legal requirements of most countries with regard to patient safety and privacy protection. It is subsequently embedded in the clinical governance rules.” [33].

Following on from Table 1 in Section 4, Table 2 below highlights the type of medicine adherence example (for asthma) from a Health 4.0 perspective. This moves beyond the pure ‘Service Managed’ model and now activates the individual user, who must act cooperatively as defined by the service for maximum gain for both user and system. In this context there is a subtext of enhanced resilience through remote medical device management—with the smart inhaler subject to remote diagnostics and possibly calibration. It also implies that there is an extra degree of security and medical device regulation / clinical governance to satisfy the life critical nature of the services.

The current Health 4.0 proposition begins to expand on the 4.0 toolset and holds clear benefit in the form of improved service and treatment quality while reducing associated transactional costs such as for medical device management, remote monitoring, and medicines adherence. However, given the policy shift towards co-managed, integrated community care, and given the sustainability challenges around the current medical model, the application of 4.0 capability requires evolution and extension.

## 6. Towards a New Paradigm: Care 4.0

Referring to the learnings identified in Section 3, co-design with people providing and receiving care provides a set of underpinning requirements for how to approach a new paradigm of care. Specifically, that technology should enable connections between people, create an environment that fosters trust, allows agency, but also recognizes assets and responsibilities as part of a wider, interdependent community. This kind of environment would allow the many informal carers and wider third sector, voluntary and independent services to be activated and participate in a trusted way, building capacity and resilience so that formal services can be a point of escalation and oversight, rather than the default point of access to care.

The following sections will analyze these requirements in the context of the 4.0 toolset, through three different lenses: (1) Comparison of different trust models; (2) a service, self and networked managed matrix approach; and (3) the context and qualities of the different applications of the 4.0 toolset.

### 6.1. Trust Models

Creating any new relationship requires a degree of trust. This is true of individual relationships, and the relationships between people and organizations, as well as between organizations. Trust can be established more quickly using a variety of methods—most typically by relating the trustee to an entity that is already trusted. 

In the context of health and social care trust can be defined predominantly in two ways. 

#### 6.1.1. Organizational Trust

The first is an organization-centric approach. Weick and Sutcliffe [43] describe ‘High Reliability Organizations’ (HROs) that have developed practice to mitigate the likelihood of catastrophes that may be expected due to the high-risk factors and complexity, for example air traffic control. The organizational properties include a preoccupation with failure, the ability to allow expertise and not hierarchy to determine safety actions, determination not to oversimplify the environment—recognizing the ‘messiness’ in complexity, and an ongoing commitment to resilience—i.e., the ability to absorb and recover from strain on the system.

Public policy makes it clear that trust in a health and social care service is perceived to be built on a combination of quality, security, safety, and resilience—i.e., the measures that can mitigate risk and create a stable and predictable service and that quality and safety are inextricably linked [12,44,45,46,47]. Within this context, there are many attempts to translate HRO properties into healthcare environments [48,49].

Nemeth and Cook [50] question the assumption that the health and care sector can achieve these HRO properties. They note a tension in the inherent need to standardize and simplify to achieve reliability in this way, which may impede rather than help in complex care scenarios. Specialized facilities (e.g., for cardiac surgery) can adopt HRO properties due to their ability to focus and optimize one particular environment and treatment and be selective about the cases they take [50]. As you move through the health and care tiers into general hospitals, then primary and community care, needs become more complex and diverse and so cannot be handled by reliability measures dependent on degrees of standardization and simplification. Further, cost limits and demand pressures make it very difficult to prioritize reliability measures in health and care in general. 

It is worth noting that there is limited evidence of a link between this kind of patient safety focused organizational culture (not measures) and improved patient outcomes [51], but links have been demonstrated between this kind of culture and improved patient experience [52].

Nemeth and Cook posit that it may be better to cultivate other aspects of health and care that support resilience, suggesting the use of information technology (IT) to improve healthcare efficiency, safety, and reliability [50]. This paper leaves open the question of how to use information communication technology (ICT) for data sharing to support trusted service delivery. There is a growing literature around security, privacy and interoperability standards, new cyber-resilience tools, questions of who grants access to data, and competence in security for patients and professionals [53].

Beyond the more controllable centralized specialist care facilities, the overall public health model is decentralized, with sustainability improvements focused on the types of services that can be delivered safely by other community ‘hubs’ with less specialism and resource, e.g., pharmacy, general practice. In turn these structured, closely governed service hubs then transact with a diverse range of more informal arm’s length services (from an acute medical perspective) provided by social care, third sector, or independent carers. Differing approaches to governance between these organizations can make it difficult to form trusted joint service models. In many cases the relationship between formal service managers and commissioners and informal care providers is strained and difficult [54,55].

#### 6.1.2. Personal Trust

The second is a person-centered approach, focused on relationships. Mayer, Davis and Schoorman [56] define trust, in the context of inter-personal relationships in an organizational setting, as “the willingness of a party to be vulnerable to the actions of another party based on the expectation that the other will perform a particular action important to the trustor, irrespective of the ability to monitor or control that other party”.

This definition contrasts the kind of trust people are looking to grant to a care giver with the governance mechanisms used by organizations to mitigate risks of poor service—legalistic remedies described as “weak, impersonal substitutes for trust, which may bring organizational legitimacy, yet often are ineffective” [56]. Mayer, Davis and Schoorman propose three qualities that appear to explain a major portion of trustworthiness. Ability is the “skills, competencies and characteristics that give a party influence in a specific domain; integrity is “the trustor’s perception that the trustee adheres to a set of principles that the trustor finds acceptable”; benevolence is “the extent to which a trustee is believed to want to do good to the trustor” [56].

This second type of trust (person-centered approach) will be required to deliver the community of care called for by co-design and the distributed, co-managed care model mandated in government policy. Furthermore, it is important because the strength of informal inter-personal networks is a part of what makes community care sustainable [57,58], and this is also another source of inherent resilience as called for by Nemeth and Cook [50].

The trust models outlined in Table 3 are not mutually exclusive. The type one trust components of quality, security, safety, and resilience are part of the ‘ability’ requirement in type two. Health and social care organizations mainly focus on type one, but also depend on professional standards bodies to ensure ability, integrity, and benevolence (type two trust) in their members. The 4.0 toolset can be deployed to support both types of trust model. However, it is anticipated that significant gains will come from the use of these capabilities to empower individuals and informal organizations to be able to demonstrate their trustworthiness, so they can be better recognized, valued and accessed in the health and social care economy. For example, peer review websites may help with ability and benevolence, while emerging distributed ledger technologies (i.e., blockchain) may help with the integrity and provenance of data or determining the authenticity of a website’s information. Individuals may become more trusting and more trustworthy if they can demonstrate ability, integrity, and benevolence, and be held accountable through these methods. 

Reflecting on the additional ‘security, safety and resilience’ design principle proposed by Thuemmler and Bai [33] for Health 4.0, this could be expanded for Care 4.0 to a ‘trust’ design principle that includes both organization and person-centric trust requirements. This would need to go beyond securing an electronic health record (EHR), which is the subject of the majority of discussion in this area [53], and this paper’s authors hope to stimulate secure but distributed trust architectures for community care.

### 6.2. Service, Self, and Network Managed Care

Throughout this paper, each application example has been accompanied by a summary table (Table 2 and Table 3) that highlights the key 4.0 capabilities and how they may influence a type of service model for the example in question. The consistent method across all three examples is ‘service managed’ in which an organization provides a service directly to an individual consumer, subject mainly to the type one trust model (Section 6.1). The Health 4.0 example developed this further, imagining the self-managing individual fulfilling their part of the clinical service to complete a feedback loop that helps the organization provide a better service for them. In this scenario the person with asthma would be consuming a service primarily in line with the type one trust model. There would be some type two trust components, but at the level of the doctor or nurse–patient relationship. This may not be as strong as it once was if the person cannot access the same individual consistently, and the trust might further be eroded as the ability of the formal asthma care system falters in the face of an increasingly informed service-user with higher expectations. 

When that person is encouraged to empower and educate themselves, they will often then develop their own methods and tools to maintain and promote their wellbeing in a broader ‘whole of life context’ [59,60,61]. This might include an app to monitor cough frequency, an air quality monitor to warn of likely triggers or a peer support webchat service. At this stage the predominantly type one trust method used by their clinical service will not be able to move with them, with their independent, third, and private sector options all posing unacceptable risks from an organization-centric trust perspective. The impulse from the clinical service would be more expensive and less consumer friendly, but more heavily regulated services and tools. The very act of making a service compliant in this way may indeed denature it of the qualities that allowed the person to use it easily and preventatively in their life in the first instance.

The 4.0 toolset could allow for a partial rationalization of these consumer services that may allow formal health and social care services to act in concert with them. It is anticipated that these tools may allow social prescribing, self-directed support, or other distribution methods to take some formal care service seed funds and use them to activate networks of individuals and informal organizations that can move more dynamically with the person. The key to this will be capabilities that build feedback loops to formal service providers around the transactions and outcomes to satisfy their type one trust requirements. The same infrastructure can also be used to create trust between individuals and informal organizations to help new service and business models emerge that trade on networks of empowered, trusted individuals. 

Table 4 (below) summarizes both the service and self-managed care methods, but then also adds a network managed method. These are not mutually exclusive concepts. There will always be the need for (and a benefit in applying the 4.0 toolset to) core centralized and decentralized medical capabilities delivered through service managed approaches. Here will always be a group of people who need to depend on this relationship and are happy to self-manage to optimize the service they are provided. This paper proposes that there is already a significantly larger component of care provided by networks of people outside of formal services, and that there is a danger that the 4.0 toolset will be applied in a way that ignores or disrupts those networks. The architecture of a ‘whole of life’ care system must be designed to empower individuals to interdepend on each other through networked ‘communities of care’. 

Future work could use Table 4 to plot how the innovation and research communities currently focus their efforts. It is anticipated that most activity and resource will cluster to the top left, i.e., the use of relatively simple telehealth technologies to provide the service, and sometimes the user decision support using data the user collects. Activities will likely then distribute along from left to right in the service managed care method, with very little in the network managed layer and next to no activity in the bottom right of the figure.

### 6.3. Comparative Context and Qualities for the 4.0 Toolset

**Figure 3:**
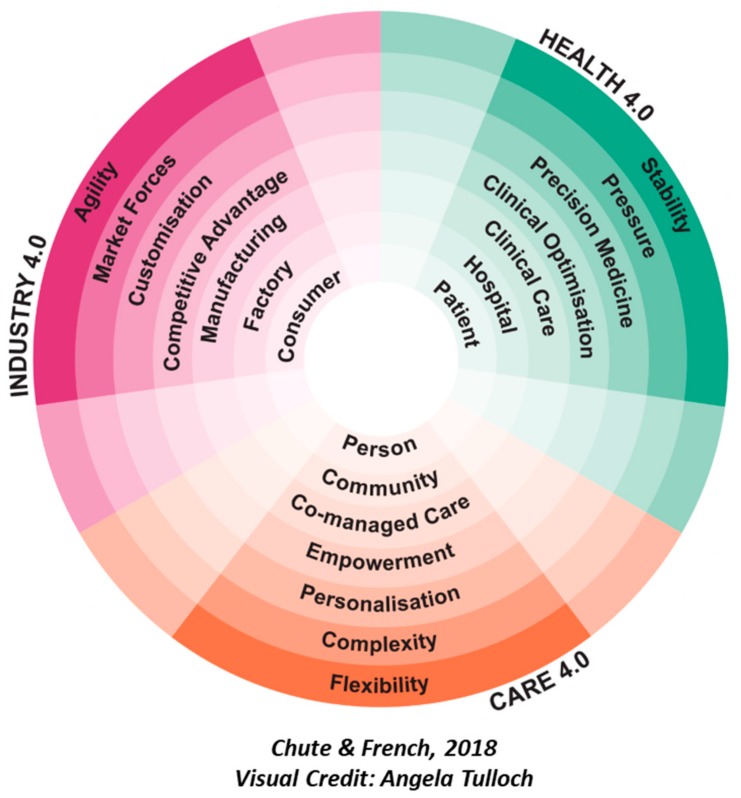
Industry 4.0 applied to new, integrated health, and social care services (Care 4.0).

Figure 3 depicts, from the center outwards, the labels, locations, processes, drivers, tailoring methods, complications, and contributions inherent in the different applications of the 4.0 toolset. For the purpose of developing the Care 4.0 concept, high level labels were created to facilitate a discussion around the difference in delivery modes between Industry, Health, and Care 4.0.

The qualities that alter the 4.0 requirements for person-centered, integrated, and co-managed care (Care 4.0) are as follows:

Role/Label—Person: The emerging policy and practice shifts which aim to place the person at the center of care requires a shift in the language used in order to reflect the various terms used to describe people who receive care, in the context of the method of care in question. Currently, these include patients (health care service managed perspective) and service users and clients (social care service managed perspective). The individual may also be an independent consumer that self-manages in the context of a relationship with a service provider (self-managed perspective). In all cases these are *role* descriptions. The proposed Care 4.0 approach views the person in totality—beyond their role in relation to an organization, including their needs and aspirations for care but also how they would prefer to interact with people, services, and organizations that can support their wider needs. This will create the environment for a more personalized and preventative approach (a mixture of service, self and network managed perspectives).

Default Location of Care—Community: Government policy is driving the system to provide care in a homely or community context. But also diversifying and recognizing who provides care—both formal and informal parts. There is the need to explore the role of the community (and a person’s specific ‘community of care’) in providing care, but discerning when this is appropriate and how people connect to other people and services. The 4.0 toolset will enable place-based and virtual communities and will unlock the environmental data that may provide context to improve a person’s care experience.

Core process—Co-managed care: Both government policy and co-design outputs highlight the importance of rebalancing power between care provider and receiver. The 4.0 toolset could help people play an active role in their care through shared decision making and potentially using their own comfortable technologies to inform this. This co-management extends to the different roles and responsibilities when networks of people provide or receive care.

Key Driver—Empowerment: Recognizing what people might be enabled to do for themselves, and how to create resilient and connected workforce and communities is a key driver. This emphasizes the need to support people to be independent and interdependent rather than dependent and taking an asset-, rather than a deficit-based approach. Within the same context, health and social care professionals also need to be empowered, with permission to be more autonomous and innovative.

Tailoring Method—Personalization: It is difficult to personalize care without the context of someone’s lived experience. Medical history does not account for wider social and life factors. Moving beyond customization to personalization, new services can be offered that are cognizant of the person’s assets, goals, and preferences, and activated through shared decision making. The 4.0 toolset creates a ‘learning system’ with largely automate feedback loops throughout the value chain so that the service can be adapted/enhanced on an ongoing basis. 

Key Complication—Complexity: Each of these paradigms has a complication, a facet of delivery that requires connected systems and automation to make the services manageable. In the case of Care 4.0, a distributed, co-managed health and social care approach is significantly more complicated that a centralized, organization-led approach. It is inherently less controllable and consistent, resources are less available and more fragmented, there are more people involved, most of whom are not employed by one organization and accountability is more difficult when so many factors influence outcomes at once. The 4.0 toolset can create a cyber-version of the physical world and a lot of its complexity. This would allow a better degree of orchestration with an overview of all the actors and factors and more ability to establish trusted links between people, organizations, and outcomes. 

Key 4.0 Contribution—Flexibility: A rigid, centralized system will not be able to keep pace with, nor handle the inherent risks of both inter-personal informal network activities, and individual consumer behaviors. Creating a distributed trust architecture will allow new forms of service to emerge that can then create the quality, security, safety, and resilience proofs required for individual and informal carers to interact productively with formal health and care organizations.

The qualities differ between applications of the 4.0 toolset. For example, the key 4.0 contribution to a hospital environment versus a community environment are markedly different with one seeking stability and one seeking flexibility. In general, supporting networks of people differs substantially from supporting an organization. These qualities provide an initial set of high-level system requirements that can be used as a framework to guide future innovation in this context. This will support organizations to recognize the inherent complexity and flexibility required in the context of user need, rather than defaulting service design to satisfy organizational requirements of risk management and trust.

## 7. Conclusions

This paper has considered Industry 4.0 as a base set of capabilities that are helping to transform many different sectors and organizations. There are many examples of the use of this 4.0 toolset emerging in medicine. This has been characterized as ‘Health 4.0’ but encompasses many related narratives around the use of the IOT in medical care. These approaches tend to focus on the patient in a hospital or other clinical setting, optimizing and tailoring treatment for better clinical outcomes, and to stabilize a stretched healthcare system. As an extension of traditional telemedical/telehealth initiatives, a broader array of 4.0 capabilities continue to attract billions of pounds of investment, research, and innovation funds globally. However, this paper has highlighted that this investment does not align well with the emerging government policy and person-centered co-design of digital health and social care services.

Learning and implications from research in the DHI in Scotland posits that there is little innovation, research and development activity focused on the application of full value chain 4.0 approaches for person-centered, distributed health and social care activity. Care 4.0, introduced in this paper, would use the same underlying 4.0 capabilities, but focus on how better-connected people and environments could help people co-manage and use their own assets, in the context of their own care circle and community. This is also built around the current relationships, individual context and use of technology in people’s everyday lives. It would enable personalized services that are more responsive to care needs and aspirations, offering preventative approaches that ultimately create a more flexible and sustainable set of integrated health and social care services that support meaningful engagement and interactions.

Operationalizing Care 4.0 requires exploration of how to use the 4.0 toolkit in the context of personal and sensitive care situations. Issues of trust, ethics, ownership and control become paramount in order to ‘humanize’ 4.0 for a person-centered care setting. Further, the distributed and complex nature of community care will necessitate 4.0 to support navigation and assistance to help people to use their data to activate services on their own terms, at the right time and in the right place.

In this and future papers a framework will be developed for researchers, innovators, and policy makers to enable exploration of digitally supported care delivery outside of the dominant discourse focused on organization-centric delivery, clinical excellence, and advanced medical technologies. Future work should more thoroughly define and identify the gaps between the paradigms of Industry 4.0, Health 4.0 and Care 4.0.

Specifically, there is a call for three types of follow up activity:

1. Investigation of trust and the brokerage services that may need to emerge to support a distributed/network model of care delivery.
2. Definition of new types of trust architecture that can automate the ability to share data, prove risk or eligibility, and generate insights without compromising a persons’ privacy and agency.
3. Redesign of existing health and social care services through co-design to inform and be informed by (1) and (2).

Through this approach we can shape a set of fundamental activities that help change the fabric of the system rather than continue to add technology in a reactive way. Success will be dependent on rebalancing the ecosystem to support care networks and co-management at scale.

## Figures and Tables

**Table 1:** Applying the Industry 4.0 toolset in manufacturing.

Owner	Cyber-Physical System	Internet of Things	Internet of Services	Smart Factory (Virtual Agent)
Service	A storage unit on a factory production line assesses capacity and orders a re-stock.	Multiple storage units aware of each other may coordinate stock orders to reduce transport costs.	Over time a company may specialize in proactive re-stocking based on the data provided by networks of connected storage units.	A factory foreman may change the work schedule if the stock systems projects a price drop for certain required components.

**Table 2:** Industry 4.0 toolset applied to asthma care.

Owner	Cyber-Physical System	Internet of Things	Internet of Services	Smart Factory (Virtual Agent)
Service Managed	A smart inhaler monitors the real world and then creates a digital (cyber) record of medicine adherence for the organization.	Inhaler, sleep and activity monitoring devices exchange data to help the organization better understand contextual factors leading to exacerbations.	The combined digital records are used by the organization to offer preventative advice, e.g., automated messages to the user: “your risk is high—use your preventer“.	A virtual agent can: - Support a doctor to personalize medicines and review appointments. - Access known triggers to enhance the model, e.g., regional air pollution. - Support the doctor’s practice by feeding back outcome data.
Self-Managed	A smart inhaler monitors the real world and then creates a digital (cyber) record of medicine adherence for the user.	Inhaler, sleep and activity monitoring devices exchange data to help the user better understand contextual factors leading to exacerbations.	The combined digital records allow the user to prove risk/eligibility and access services. Third parties can offer the user advice on sleep quality to reduce risk.	A virtual agent can: - Support a user to personalize their own care plan based on previously unknown triggers. - Connect the user to other services and communities to help them access broader support.

**Table 3:** Trust model comparison.

Type 1: Organization-Centric	Type 2: Person-Centric (Organizational Context)
Trust = Quality, Security, Safety, Resilience	Trust = Ability, Integrity, Benevolence
Trust between a user and formal services	Trust between people
Trust between formal service providers	Trust between formal and informal services

**Table 4:** The 4.0 toolset applied across care organization.

Care Method	Cyber-Physical System	Internet of Things	Internet of Services	Smart Factory (Virtual Agent)
Service Managed	A single device monitors the real world and then creates a digital (cyber) record for the organization.	Several devices combine their digital records to help the organization better understand context and create insight.	The digital records are used by the organization to offer new services. New business models are possible because the organization has live information, so it can personalize services and manage risk better. New business models emerge for joint benefit between organization and user.	A virtual agent can: - Support a worker to personalize the service based on the digital records. - Access global data sets to finesse the service by understanding and predicting needs and issues. - Support the end-user to understand outcomes, then tailor future activities.
Self-Managed	A single device monitors the real world and then creates a digital (cyber) record for the user.	Several devices combine their digital records to help the user better understand context and create insight.	The digital records are used by the user to activate new services. New business models are possible because organizations can be given data, so that they can personalize services and manage risk better. New business models emerge for direct service to a user.	- Support a user to personalize their own service based on the digital records. - Access global data sets to tailor the service by understanding and predicting needs. - Support the user to understand outcomes and modify the activities of user and organization.
Network Managed	A single device monitors the real world and then creates a digital (cyber) record for the user and a network of people and organizations they trust.	Several devices combine their digital records to help the user and their trusted network to better understand context and create insight.	The combined digital records are used by the user and their trusted network to activate new services. New business models are possible because organizations can be given data/trend information, so that they can personalize services and manage risk better. New business models emerge between groups of organizations and users.	- Support groups of users to access peer support and personalize their own service based on the digital records. - Create new data sets to understand group needs. - Access global data sets to finesse the ability to predict needs and issues. - Support groups of users to understand outcomes and then modify the user, group and organization’s future activities. - New business models emerge between groups of users/organizations.
